# Supplementary figures and images for: Presence of Cutaneous Complement Deposition Distinguishes between Immunological and Histological Features of Bullous Pemphigoid—Insights from a Retrospective Cohort Study
Source: J Clin Med. 2020 Dec 3;9(12):3928. doi: 10.3390/jcm9123928 (PMC7761814; doi:10.3390/jcm9123928)

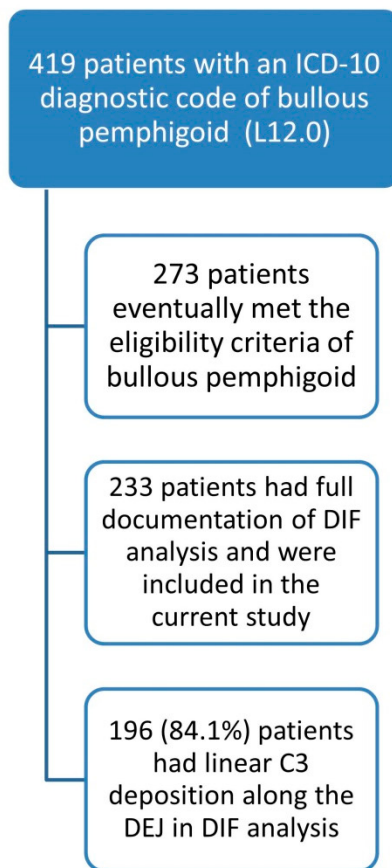

**Figure 1.** Flowchart clarifying the selection of study population.

Supplement: Supplementary file 1 [file jcm-09-03928-s001.pdf]
